# Telomeres and atherosclerosis

**DOI:** 10.5830/CVJA-2012-056

**Published:** 2012-11

**Authors:** Sajidah Khan, DATSHANA P NAIDOO, Anil A Chuturgoon

**Affiliations:** Department of Cardiology, Nelson R Mandela School of Medicine, University of KwaZulu-Natal, Durban, South Africa; Department of Cardiology, Nelson R Mandela School of Medicine, University of KwaZulu-Natal, Durban, South Africa; Discipline of Medical Biochemistry, Nelson R Mandela School of Medicine, University of KwaZulu-Natal, Durban, South Africa

**Keywords:** coronary artery disease, molecular and cellular cardiology

## Abstract

**Abstract:**

In humans and other multicellular organisms that have an extended lifespan, the leading causes of death are atherosclerotic cardiovascular disease and cancer. Experimental and clinical evidence indicates that these age-related disorders are linked through dysregulation of telomere homeostasis. Telomeres are DNA protein structures located at the terminal end of chromosomes and shorten with each cycle of cell replication, thereby reflecting the biological age of an organism. Critically shortened telomeres provoke cellular senescence and apoptosis, impairing the function and viability of a cell. The endothelial cells within atherosclerotic plaques have been shown to display features of cellular senescence. Studies have consistently demonstrated an association between shortened telomere length and coronary artery disease (CAD).

Several of the CAD risk factors and particularly type 2 diabetes are linked to telomere shortening and cellular senescence. Our interest in telomere biology was prompted by the high incidence of premature CAD and diabetes in a subset of our population, and the hypothesis that these conditions are premature-ageing syndromes. The assessment of telomere length may serve as a better predictor of cardiovascular risk and mortality than currently available risk markers, and anti-senescence therapy targeting the telomere complex is emerging as a new strategy in the treatment of atherosclerosis. We review the evidence linking telomere biology to atherosclerosis and discuss methods to preserve telomere length.

## Abstract

Atherosclerosis is an age-related disorder.^1^ Premature biological ageing, an entity separate from chronological ageing, may contribute to its pathogenesis. Cellular senescence, which is defined as the finite replicative lifespan of cells leading to irreversible growth arrest, plays a critical role in the pathogenesis of atherosclerosis.^2^-^4^ A central feature of atherosclerosis is vascular endothelial cell dysfunction.

The histology of atherosclerotic plaques has been comprehensively studied and has demonstrated that endothelial and vascular smooth muscle cells in atherosclerotic lesions display changes of senescence.^5^,^6^ In stable atherosclerotic plaques there are few senescent cells, whereas in advanced, complicated plaques, senescent cells accumulate because of high cell turnover and increase the risk of acute coronary syndromes.^7^ The biological mechanism that triggers the onset of cellular senescence is thought to be telomere shortening.

Telomeres are DNA protein structures located at the extreme ends of the chromosomes.They cap and protect the ends of chromosomes. Whereas the DNA molecule carries the genetic code and is about 100 million base pairs long, the telomeric ends are non-coding and are between 5 000 and 15 000 base pairs long: 15 000 at the time of human conception and around 5 000 at the time of death.^8^

During DNA replication, the very end sequences of the telomere are not fully copied due to the inability of DNA polymerase to completely replicate the chromosome to its very end. This is termed the end-replication problem. As a result, between 50 and 200 nucleotides are lost with each cycle of cell replication, leading to progressive telomere shortening.^9^ When telomere length reaches a critical threshold, the cell becomes incapable of further replication and enters a phase of cellular growth arrest termed replicative senescence. On average, cells reach senescence after 50 divisions. The senescent phase may then progress to cell death or apoptosis.

Cellular senescence and the apoptotic cascade are mediated by cell cycle checkpoint pathways, regulated mainly by p53/p21, which are best recognised as tumour suppressor proteins.^2^ This process is responsible for physiological ageing and gives rise to the morphological and functional changes that accompany the decline in organ function seen with age, e.g. endothelial cell senescence in atherosclerotic plaques or beta-cell senescence in diabetes mellitus.^4^,^10^,^11^

However, a limited number of cells (about one in 10 million) are able to reactivate the enzyme telomerase. In the presence of telomerase, cells are able to replicate and in this way telomere integrity is maintained. Telomerase activity is lacking in somatic cells but is preserved in reproductive and stem cells. High telomerase activity has also been detected in about 90% of human cancer samples. The high telomerase activity is thought to be responsible for the indefinite cell proliferation and cellular immortalisation seen with cancer.^12^-^15^ Inducing cell senescence and apoptosis is therefore an important mechanism for the suppression of cancer.

Studies have shown that telomere length is not only determined by cell replication and lifespan, but is also influenced by heredity and exposure to environmental risk factors. The healthy offspring of parents with coronary artery disease have shorter telomeres than the offspring of normal subjects.^16^,^17^ The traditional risk factors for atherosclerosis have been shown to lower the threshold for cardiovascular disease by hastening biological aging.^18^ Risk factors such as smoking,^19^,^20^ obesity,^19^ insulin resistance,^21^,^22^ and type 2 diabetes^23^-^26^ are associated with accelerated telomere shortening.

Diabetic patients, more than any other subset, show the greatest difference in telomere length compared to non-diabetics.^26^ Type 2 diabetes is considered a cardiovascular risk equivalent.^27^,^28^ It is postulated that telomere shortening induces pancreatic β-cell senescence. Like atherosclerosis, diabetes is thought to be a premature-ageing syndrome.^26^

The study of telomeres may therefore provide in a single marker, the combined influence of genetics, environmental risk and ageing in predicting risk and identifying susceptible individuals prone to developing coronary artery disease. This is especially relevant in our community, which has a high incidence of both premature coronary artery disease and type 2 diabetes.^29^,^30^

## Structure and function of the telomere complex

**Fig. 1:**
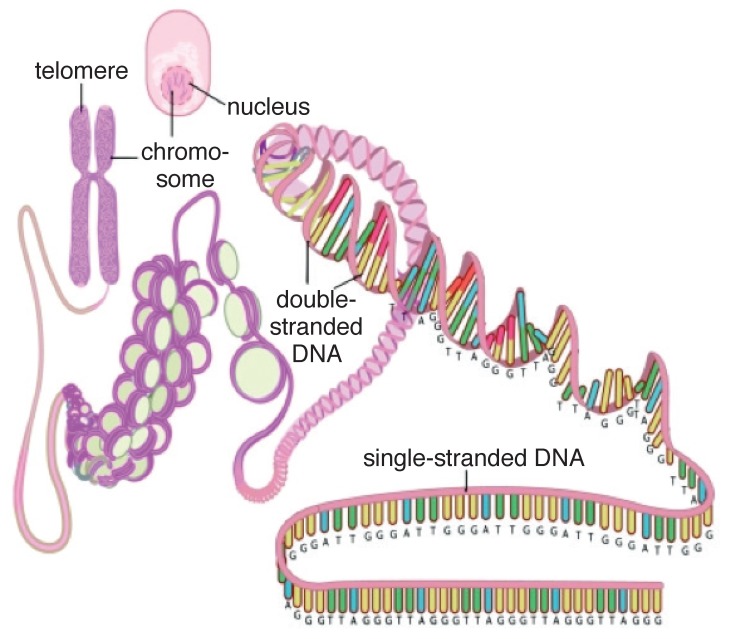
A simplified scheme depicting the structure of the telomere and its location on the chromosome in the cell. Reproduced with permission.^126^

**Fig. 2:**
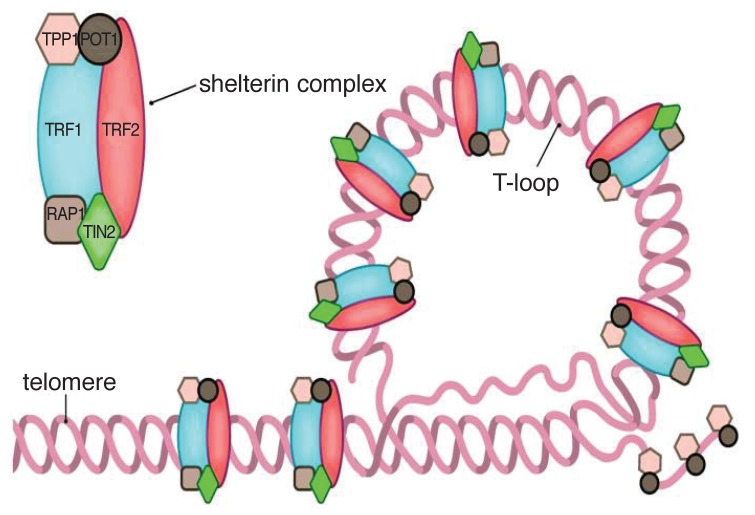
Scheme showing the terminal end of the telomere concealing the terminal single-stranded part with the help of the shelterin complex. Reproduced with permission.^126^

Telomeres have a dynamic structure that is thought to switch between a closed, protected state and an open, extendable state, which allows the DNA terminus to undergo replication. The protected state is necessary for safeguarding the integrity of genomic material, whereas the extendable state allows the enzyme telomerase to extend short telomeres (Figs 1, 2).^31^ Telomere components include:

• The DNA component: this consists of tandem repeats of the hexanucleotide 5′-TTAGGG-3′ (T = thymine, A = adenine, G = guanine) and has a high guanine content. The bulk of telomeric DNA is arranged in the double-stranded configuration, which then ends in a single-stranded extension. The single-stranded overhang folds back to form a terminal loop, which prevents the end of the telomere from being recognised as a damaged, broken end. Telomere shortening is thought to destabilise this loop.^8^,^14^,^31^
• Shelterin proteins: these proteins bind and protect the loop structure and are termed shelterin because they shelter the chromosome end.32 An inability to form the terminal loop will leave the chromosome ends uncapped, resembling a DNA break and provoking DNA repair mechanisms. The shelterin complex consists of six proteins, which have specific functions in telomere replication and end protection.
The six proteins are: TRF1 and TRF2: telomere repeat-binding factors 1 and 2, which are the two major proteins; POT1: protection of telomeres 1; TPP1: tripeptidyl peptidase 1; TIN2: TRF1-interacting protein 2; and RAP1: repressor activator protein 1. Whereas the shelterin proteins are a constant fixture at the telomere end, other accessory proteins are intermittently recruited to the telomere. These proteins include the tankyrases tank 1 and 2, Ku 70/86 and poly-ADP ribose polymerase-1 (PARP-1), which influence the control of telomere length and repress the DNA damage response.^31^,^33^,^34^
• The CST complex: an additional telomere-associated complex, known as the CST, has recently been identified. It binds single-stranded DNA and appears important for both telomere protection and replication.^31^
• Telomerase: in order for cellular repair to take place as well as for species survival, stem cells and reproductive cells need to be able to proliferate without the penalty of progressive telomere shortening.^31^ These cells, unlike somatic cells, contain the enzyme telomerase, which is capable of adding DNA sequences to the chromosome terminus to compensate for the loss sustained during replication. Telomerase is made of Terc – the RNA component that serves as a template for the synthesis of new telomeric DNA, and TERT – a reverse transcriptase which is the catalytic subunit representing the rate-limiting step in telomerase activity.^12^,^14^,^33^,^35^ A variety of accessory proteins have important roles in telomerase biogenesis and localisation.

## Telomere homeostasis

Telomere length in proliferating cells is influenced by the following factors.

• Factors that shorten telomeres:
- telomere attrition during cell division
- DNA damage due to oxidative stress caused by environmental risk factors
- specific exonucleases involved in the degradation of RNA primers used for DNA replication
- deficiency of Rad 54, which is involved in DNA repair
- histones: methylation of histones H3 and H4 diminishes telomerase activity.^36^
• Factors that maintain telomere length:
- Telomerase: in addition to the level of telomerase within a cell, telomere length is also dependent on the delivery of telomerase to the telomere by Cajal bodies, telomerase access to the DNA terminus and the presence of molecules that stimulate or inhibit telomerase activity.^31^
- A recombination process known as alternative lengthening of telomeres or ALT (10% of cancers maintain their telomere length by ALT).^35^,^37^

The two major mechanisms responsible for telomere shortening are the end-replication problem, and more importantly, the oxidative DNA damage induced by environmental risk factors. Telomere shortening due to the end-replication problem is relatively small and constant in each cell, irrespective of telomere length, whereas telomere shortening induced by oxidative stress is proportional to telomere length, as longer telomeres are larger targets for free radicals.^38^-^40^

Variability in telomere length is also noted at birth and is influenced by heredity, race and gender. Telomere length has been shown to be shorter in healthy offspring of patients with coronary artery disease (CAD).^16^,^17^ This finding offers some explanation for the increased familial risk of CAD and also implies that shorter telomeres are likely a primary abnormality in the pathogenesis of the disease.^41^ African-Americans have longer telomeres than whites and Indians,^42^-^44^ and females have longer telomeres than their male counterparts.^45^

## Mechanisms of disease: a balance between injury and repair

## Mechanism of injury: oxidative stress

Oxidative stress is the unifying pathophysiological mechanism responsible for ageing and age-related disorders.^46^-^49^ It is defined as an increase in the intra-cellular concentration of reactive oxygen species (ROS). ROS are generated during regular metabolism because of incomplete oxygen reduction in the mitochondrial electron transport chain – a one-electron reduction of oxygen forms superoxide (O_2_^-^), a two-electron reduction forms hydrogen peroxide (H_2_O_2_), and a three-electron reduction forms the hydroxyl radical (OH). Many other ROS species can be derived from superoxide and hydrogen peroxide.

These ROS initiate processes involved in atherogenesis through several enzyme systems including xanthine oxidase, NADPH (nicotinamide adenine dinucleotide phosphate) oxidases and nitric oxide synthase.^50^ The ROS damage all components of the cell including proteins, lipids and DNA. The exact mechanism of damage is via:

• Decreased availability of nitric oxide (NO), which results in defective endothelial vasodilation. Nitric oxide is an antiatherosclerotic agent that protects vascular cells from apoptosis.^51^-^53^
• Inflammation: ROS increase the production of pro-inflammatory cytokines such as tumour necrosis factor alpha (TNF-α), which in turn can also increase the production of ROS. TNF-α activates two transcription factors: nuclear factor kappa-β (NF-κβ) and activator protein-1 (AP-1), which increase the expression of pro-inflammatory genes. Cytokines stimulate the synthesis of acute-phase reactants such as C-reactive protein (CRP) by the liver. ROS also increase the expression of cellular adhesion molecules on the endothelial cell surface. These molecules, intercellular adhesion molecule 1 (ICAM-1) and vascular cell adhesion molecule 1 (VCAM-1), enhance monocyte adhesion to endothelial cells and lead to the formation of atherosclerotic plaques.^54^-^58^
• Modification of lipoproteins and lipids: ROS contribute to the formation of lipid peroxides, which bind to proteins to form advanced lipoxidation end products (ALEs).^59^ Oxidised LDL and ALE-containing LDL are pro-atherogenic. *In vitro* studies have shown that LDL cholesterol (LDL-C) is not atherogenic in itself but it is the oxidative modification of LDL-C that plays a critical role in the pathogenesis of atherosclerosis.^60^,^61^ In the early phase of atherosclerosis, oxidised-LDL (ox-LDL) contributes to inflammation by enhancing expression of chemokines such as the monocyte chemo-attractant protein-1. Ox-LDL decreases the bioavailability of nitric oxide. The proatherogenic effects are exerted by influencing the phosphoinositol-3 (PI3) kinase/Akt signalling pathway.^62^

This pathway has an important regulatory role in cellular proliferation and survival. Of the three known isoforms of Akt, Akt 1 is most relevant in regulating cardiovascular cell growth and survival and Akt 2, which is highly expressed in muscle and adipocytes, contributes to regulation of glucose homeostasis. These isoforms are activated by growth factors, extra-cellular stimuli such as pro-atherogenic factors and by oncogenic mutations in upstream regulatory proteins. Akt mediates downstream signalling pathways through phosphorylation of a host of substrates. Thus far, more than a hundred substrates for Akt have been identified, indicating that it has widespread biological effects. Dysregulation of Akt is associated with cardiovascular disease, diabetes, cancer and neurological disorders.

Our current understanding of its role in cardiovascular disease is incomplete and studies explaining its effects describe conflicting mechanisms. Breitschopf *et al.* have demonstrated that pro-atherogenic factors such as ox-LDL, TNF-α and hydrogen peroxide promoted endothelial cell senescence by inactivation of the PI3/Akt pathway. Akt was shown to maintain telomerase activity by phosphorylation of its TERT subunit, and inactivating Akt reduced telomerase activity, leading to accelerated endothelial cell senescence.^63^

On the other hand, Miyauchi *et al.* demonstrated that activation of Akt promotes senescence and arrests cell growth via the p53/p21-dependent pathway and that inhibition of Akt extends the lifespan of primary cultured human endothelial cells. Akt achieved growth arrest by phosphorylating and inhibiting a forkhead transcription factor (FOXO 3a), which influences p53 activity by regulating levels of ROS.^64^ Rosso *et al.* confirmed the latter mechanism by demonstrating that endothelial progenitor cells cultured in the presence of ox-LDL in a diabetic milieu underwent senescence and growth arrest by activation of the Akt pathway via accumulation of p53/p21.^65^

Miyauchi *et al.* commented that the divergent observations may be explained by the different cell types used in studies. They used primary human endothelial cells, whereas most other studies examined immortal cells in which the normal cell cycle machinery may have been impaired. In addition, Akt may promote cell proliferation or senescence depending on other factors such as the duration and extent of its activation. It has been noted that activation of Akt in itself is insufficient to cause cancer unless combined with other oncogenic stimuli.

There is currently much interest in the development of Akt inhibitors for the treatment of cancer and it remains to be seen what effects such therapy would have on the cardiovascular system. In addition to Akt signalling, mitogenic stimuli may activate Ras signalling, which has also been shown to participate in the divergent processes of both cell proliferation and senescence.^66^

## Oxidative stress and telomere shortening

Exposure of DNA to oxidative stress produces higher levels of stress biomarkers in telomere sequences than in non-telomere sequences. 8-oxodG (8-oxo-7,8-dihydro-2-deoxyguanosine) is a sensitive biomarker for oxidative stress on DNA. Progressive increases in 8-oxodG have been shown to correlate with decreasing telomere length. The high guanine (-GGG) content of telomeres makes them particularly sensitive to damage by oxidative stress.^47^,^67^

This site specificity for guanine is due to several reasons. Firstly, guanine is the most easily oxidised DNA base as its oxidation potential is lower than that of the other three bases (adenine < cytosine < thymine). A second factor is the distribution of electrons on the DNA base. The highest occupied molecular orbital that accommodates electrons with the greatest energy determines the reactivity of DNA bases. Many of these electrons are located on the 5′-G of the GG sequence and therefore this guanine is more likely to be oxidised.

A third reason is that the ROS have different redox potentials, which may determine site specificity. For example, the free hydroxyl radicals cause DNA damage without a marked site specificity, whereas the benzoyloxyl radicals specifically cause damage to the 5′-G in GG sequence.^68^-^70^ In addition to the direct effects of ROS, telomeres, unlike the rest of the genome, appear less efficient in repairing oxidative damage.^71^ An important consequence of oxidative stress is the initiation of an inflammatory response.

## Inflammation and telomere shortening

Chronic systemic inflammation is responsible for an increase in peripheral white blood cell turnover, which in turn leads to an exaggerated telomere attrition rate.^55^ The increased white cell consumption induces haematopoietic stem cells to divide, thereby shortening their telomere length as well. Exposure to TNF-α also reduces telomere length by negative regulation of telomerase activity.^57^

DNA sampling for telomere length quantification is generally sourced from circulating white blood cells rather than human vascular tissue. It has been suggested that white blood cell telomere attrition is a consequence of systemic inflammation rather than being indicative of vascular endothelial cell ageing. The study by Wilson *et al.* demonstrated that telomere attrition in circulating blood leucocytes reflects similar changes in the vasculature and is an acceptable surrogate for vascular ageing in population studies.^72^

## Mechanisms of repair: stem cells and endothelial progenitor cells

The atherosclerotic process is characterised by endothelial cell dysfunction. Repair of the endothelium is dependent on the presence of endothelial progenitor cells, which migrate to sites of vascular injury to initiate repair. Endothelial progenitor cells are produced by haematopoietic stem cells, which, due to their higher telomerase activity, have a greater proliferative capacity. Exhaustion of the progenitor cell or stem cell pool is an important factor in endothelial cell dysfunction. Telomere length in haematopoietic stem cells (HSC) is a reflection of progenitor cell reserves, and shortened telomere length in these cells is indicative of diminished reparative capacity.^41^,^42^

The onset of atherosclerotic disease is therefore dependent on the balance between injury and repair of the endothelium – injury from oxidative stress and inflammation, and repair, which depends on haematopoietic stem cell reserves, as reflected by HSC telomere length.^41^

## Telomeres and atherosclerosis risk factors

## Smoking

Cigarette smoking is associated with increased oxidative stress.^73^ Although there is variability in the findings of different epidemiological studies, the following studies recorded an association between smoking and telomere shortening. Nawrot *et al.*, reporting on the Flemish study on environment, genes and health outcomes, found shorter telomeres in smokers compared to non-smokers.^45^ The study by Valdes *et al.* showed that women who had never smoked had longer telomeres than former smokers, and both had longer telomeres than current smokers (531 never smokers, 369 ex-smokers and 203 current smokers).

They also demonstrated a dose-dependent relationship between smoking and telomere shortening. Each pack-year smoked was equivalent to the loss of an additional five base pairs of telomere length, or 18% of the average annual loss in telomere length, compared to the rate in the overall cohort.^19^ The dose-effect relationship was subsequently replicated by Morla *et al.* who studied a cohort of male smokers with and without chronic obstructive pulmonary disease (50 smokers, 26 never smokers) in whom telomere shortening correlated with cumulative exposure to tobacco smoking.^20^

## Hypertension

Since systolic blood pressure rises with age, and diastolic blood pressure plateaus, Jeanclos *et al*. postulated that arterial pulse pressure may correlate with biological age. Among 49 twin pairs (mean age 37 years) in the Danish Twin Register, they showed a significant inverse correlation between pulse pressure and telomere length, i.e. wider pulse pressure was associated with shorter telomere length.^74^

The Framingham Heart Study found shorter telomere lengths in hypertensive males (*n* = 171) compared to their normotensive peers (*n* = 156) but the shorter telomere length was largely due to insulin resistance.^21^ Benetos *et al.* examined the relationship between telomere length and carotid artery atherosclerosis in 163 treated hypertensive males and found that telomere length was shorter in hypertensive men with carotid plaques compared to hypertensive men without plaques.^75^

## Obesity

Increased caloric intake and obesity are recognised to shorten lifespan. Adipose tissue is not only a source of ROS and pro-inflammatory cytokines but also secretes a host of bioactive molecules including angiotensinogen, leptin, resistin, adiponectin and PAI-1, which influence the function and structural integrity of the cardiovascular system.^76^,^77^ These adipocytokines influence glucose metabolism, blood pressure regulation, lipid metabolism, the coagulation system and endothelial function to accelerate the process of atherosclerosis.

Obesity is strongly associated with cardiovascular disease and promotes the clustering of risk factors such as dyslipidaemia, hypertension, diabetes and the metabolic syndrome. Obese individuals experience substantially elevated morbidity and mortality from all forms of cardiovascular disease.^78^,^79^

A retrospective analysis of the Bogalusa Heart Study examined the relationship between weight change and telomere dynamics over a period of 10 to 12 years in 70 young adults. The study showed that weight gain was associated with accelerated telomere attrition and that a rise in insulin resistance accounted for the relationship between the increase in body mass index (BMI) and telomere attrition rate.^22^

In the study by Valdes *et al.* of 1 122 healthy adult female twins (45 monozygotic and 516 dizygotic pairs, mean age 47 years), it was found that the telomeres of obese twins were 240 base pairs shorter than those of the lean sibling. The difference in telomere length between the lean and the obese corresponded to 8.8 years of ageing.^19^ The study also suggested that the mechanism by which obesity affects telomere length is through increased leptin levels rather than BMI *per se*.

Obesity is associated with high serum concentrations of leptin, which is linked to NF-κB activation, a mediating factor in the production of ROS and inflammatory cytokines.^80^ Nordfjall *et al*. confirmed the negative association between BMI and telomere length but in their study, this finding applied only to female participants.^81^

## Insulin resistance

Insulin resistance is pro-atherogenic and increases the risk of CAD even without the presence of hyperglycaemia.^82^ The mechanisms involved in atherogenesis include both systemic effects such as dyslipidaemia, hypertension and a pro-inflammatory state as well as direct effects on vascular endothelial cells, smooth muscle cells and macrophages. These three cell types have insulin receptors and effects are mediated via down-regulation of insulin signalling pathways such as the Akt pathway.

In early atherosclerosis, insulin resistance causes decreased nitric oxide production and an increase in VCAM-1, which are responsible for impaired vasodilation and inflammation. In advanced plaques, insulin resistance triggers apoptosis of cells via the Akt pathway.^83^-^86^ Apoptosis of smooth muscle cells causes fibrous cap thinning, whereas apoptosis of macrophages leads to plaque necrosis, both being pathological processes that precipitate acute coronary syndromes.

## Diabetes

In the setting of type 2 diabetes, insulin resistance and hyperglycaemia have additive effects that accelerate the process of atherosclerosis. Hyperglycaemia is associated with the activation of several molecular pathways that include the production of advanced glycation end products (AGEs),^87^,^88^ activation of protein kinase C, increased activity of both the polyol as well as the hexosamine pathways.^89^,^90^ These pathways are interdependent and induce cellular damage through the final common mechanism of increased oxidative stress.

It is well established that hyperglycaemia, even in the pre-diabetic state, induces oxidative stress^91^-^94^ and ultimately leads to cellular senescence. Cellular senescence and apoptosis occur not only in vascular endothelial and smooth muscle cells but in multiple cell lines, including endothelial progenitor cells.^95^,^96^ Type 2 diabetes can therefore be considered a premature-ageing syndrome.

In recent years several cross-sectional clinical studies have been published that demonstrate an association between shorter telomere length and type 2 diabetes (T2D).^23^-^26^,^97^-^99^ The studies suggest that there is a gradation in the severity of telomere shortening. Shorter telomere lengths were noted in patients with impaired glucose tolerance compared to controls, even shorter lengths in those with diabetes, and the shortest lengths were observed in patients with the combination of pre-diabetes/diabetes and atherosclerotic vascular disease, compared to those with diabetes or cardiovascular disease alone.^100^

Satoh *et al*. showed that CAD patients with the metabolic syndrome had shorter telomeres than CAD patients without the metabolic syndrome.^97^ Adaikalakoteswari *et al.* found that among diabetic patients, those with atherosclerotic plaques had shorter telomeres.^98^ The study by Olivieri *et al.* demonstrated that diabetic patients with myocardial infarction had shorter telomeres than diabetic subjects without myocardial infarction,^99^ and the study by Salpea *et al.* showed that among diabetic subjects, those with CAD had significantly shorter telomeres.^26^

Based on these observations, it has been postulated that critically shortened telomeres, due to a combination of inherited short telomeres and oxidative stress-induced telomere attrition, caused by the common risk factors between diabetes and cardiovascular disease, indicates greater cellular ageing in vascular endothelial cells and pancreatic beta-cells, and may be a useful biomarker of tissue ageing and disease progression.^100^

## Atherosclerosis and coronary artery disease

Minamino *et al*. have shown that endothelial cells with characteristic features of senescence are present in atherosclerotic regions of human coronary arteries. They demonstrated that inhibiting telomere function induced senescence in endothelial cells, whereas introducing telomerase suppressed senescence and extended the lifespan of these cells.^3^

Ogami *et al*. have shown that the telomeres of coronary endothelial cells were shorter in patients with CAD compared to age-matched subjects without CAD and that in the CAD patients, telomere length was shorter in endothelial cells at atherosclerotic sites compared to non-atherosclerotic sites.^101^ Chang and Harley have shown that endothelial cells in regions of the vascular tree that are subjected to greater haemodynamic stress demonstrated more pronounced telomere attrition than endothelial cells from areas with less shear stress. For example, telomere attrition rate in the iliac arteries was –147 base pairs per year compared to the internal mammary arteries at –87 base pairs per year.^102^

Okuda *et al.* also demonstrated that telomere attrition was higher in the intima of the distal abdominal aorta compared to the proximal abdominal aorta, again indicating that areas of the vasculature that undergo greater shear wall stress have higher cellular turnover rates and consequently shorter telomere length.^103^ This variable telomere attrition rate indicates the significant impact of environmental stress on telomere length.

Population studies have demonstrated a link between telomere length and CAD.^104^,^105^ In the pioneering study by Samani *et al.* of 10 cases and 20 control subjects, it was observed that mean telomere length was significantly shorter in patients with severe triple-vessel CAD compared with matched subjects who had normal coronary angiograms.^106^

A retrospective registry analysis of 383 patients (203 cases, 180 controls) showed that patients with premature myocardial infarction had significantly shorter mean telomere lengths. In this study the difference in telomere length between cases and chronologically age-matched controls demonstrated a biological age gap in excess of 11 years. Compared with subjects in the highest quartile for telomere length, the risk of myocardial infarction was increased between 2.8- and 3.2-fold in subjects with shorter-than-average telomeres.^107^ In another study of 143 normal blood donors over the age of 60 years, it was shown that subjects with shorter telomeres had poorer survival, with a 3.18-fold higher mortality rate from heart disease.^108^

In a sub-study of the West of Scotland Primary Prevention Study (WOSCOPS) that compared telomere lengths at recruitment in 484 individuals who went on to develop coronary heart disease events with those from 1 058 age-matched controls who remained free of CAD, it was shown that subjects with shorter telomere length at the time of recruitment had a significantly higher risk of developing subsequent coronary heart disease.^109^ In a case-control sub-study of the Cardiovascular Health Study that examined 419 older subjects, it was found that individuals 73 years or younger had a threefold increased risk of myocardial infarction and stroke for each one kilobase decrease in telomere length.^110^

Farzaneh-Far *et al.* measured telomere length in 780 patients with stable angina in a prospective cohort study. During a mean follow up of 4.4 years, shorter telomere length was significantly associated with all-cause mortality, independent of age, clinical and echocardiographic variables.^111^ Zee *et al*. using samples collected at baseline in the prospective Physician′s Health Study from a cohort of 14 916 initially healthy men, of whom 337 went on to develop myocardial infarction, demonstrated that participants with shorter telomere length at baseline had a significantly increased risk of incident myocardial infarction compared to age- and smoking-matched controls who remained free of vascular disease over a mean follow up of 3.85 years.^112^

Finally, in the prospective population-based Bruneck study, baseline telomere length was a significant risk predictor for subsequent myocardial infarction and stroke, independent of standard risk factors. Of note in this study was that telomere length was strongly associated with advanced pathology and acute vascular syndromes but not early atherosclerosis.^7^

## Mechanisms to preserve telomere length

Telomerase has been shown to be activated by lifestyle choices that include a healthy diet, stress relief through meditation, chronic high-intensity aerobic physical exercise as well as by pharmacological agents.^113^,^114^

Exercise has been associated with improved cardiovascular health and longevity. La Rocca *et al.* in a recent study have shown that maintaining high levels of aerobic fitness preserved telomere length.^115^ They examined young and old individuals and compared sedentary subjects who exercised fewer than two days per week for less than 30 min per day with active ones who had exercised five days per week for more than 45 min per day for five years. Telomere length was preserved in the older adults who performed chronic, vigorous exercise and was positively correlated with maximum aerobic capacity as assessed by higher VO_2max_ levels.

The molecular mechanisms exploring the protective effects of exercise on the heart has been studied in experimental animals. Exercise has been shown to promote cell survival by increasing the activity of telomerase and the expression of TRF2. The up-regulation of telomerase was mediated via insulin-like growth factor 2 and endothelial nitric oxide synthase. Exercise was also shown to decrease levels of markers of cellular growth arrest and apoptosis, such as p16, cell cycle-checkpoint kinase 2 and p53.

Molecules that enhance low residual telomerase activity or re-express silenced telomerase may help preserve telomere length. Natural products such as derivatives from the Chinese Astragalus plant, *Ginko biloba* and resveratrol have been shown to activate telomerase, the latter two via PI3k/Akt signalling pathways. The anti-oxidants *N*-acetylcysteine and α-tocopherol enhance telomerase activity.^116^,^117^ Farzaneh-Far *et al.* demonstrated in a prospective study of patients with stable CAD, an inverse relationship between baseline blood levels of marine omega-3 fatty acids and the rate of telomere shortening over five years.^118^

Aspirin, ACE inhibitors and particularly statin therapy have been shown to positively impact on the vascular endothelium via anti-senescence effects. Over and above its anti-thrombotic and anti-inflammatory effects, aspirin has been shown to decrease the formation of dimethylarginine, an endogenous inhibitor of nitric oxide synthase, thereby reducing oxidative stress and delaying endothelial cell senescence.^119^ ACE inhibitors, particularly those containing the sulfhydryl group, have been shown to delay endothelial cell senescence by activating Akt phosphorylation, increasing the expression of nitric oxide synthase and up-regulating telomerase.^120^

Several studies have suggested that the survival benefit attributed to statin therapy may be linked to its effects on telomere biology. Spyridopoulos *et al.* have shown that statins enhance the migratory capacity of endothelial progenitor cells by up-regulation of TRF2, the telomere-binding protein that stabilises telomere structure at the t-loop.^121^ Satoh and co-workers demonstrated that intensive statin therapy over 12 months, through its anti-oxidant effects, prevents endothelial progenitor cell telomere erosion in patients with CAD.^122^ A recent publication by Saliques *et al.* who studied patients presenting with acute myocardial infarction, showed that prior statin therapy was independently associated with significantly longer telomere length in subjects below the age of 64 years.^123^

## Conclusion

Our interest in telomere biology stems from the high incidence of both premature CAD and type 2 diabetes mellitus witnessed in the population that we serve. Patients with CAD who have diabetes have worse outcomes than those without diabetes. This, coupled with the fact that the initial presentation in a substantial majority of our young patients is with myocardial infarction, which carries a worse prognosis than stable CAD, further contributes to adverse long-term outcomes. Indications are that revascularisation procedures are not as efficacious in this population.

The availability of quantitative polymerase chain reaction, which is a simpler, less labour-intensive and cheaper method requiring smaller quantities of DNA compared to the standard method of southern blot analysis, has made it feasible for us to determine telomere length in our patients.^124^,^125^

The study of telomere dynamics may serve several functions. Firstly, measuring telomere length in the early years of life may indicate a genetic predisposition and help target susceptible individuals. Studies on the genetic contribution to premature CAD with genome-wide association scans have yielded little thus far, whereas an assessment of telomere length provides a more universal insight into the genetics of CAD.

Secondly, telomere length is a measure of cumulative DNA damage from multiple environmental risk factors over an individual’s lifespan and is likely a better predictor of CAD than the currently available risk markers, which are single, point measurements in time.

Thirdly, although the development and progression of atherosclerosis occurs over decades, the process is clinically silent until the manifestation of full-blown disease. The rate of telomere shortening is accelerated prior to the onset of clinical disease, so longitudinal assessments of telomere length may be of predictive value. Finally, novel therapies aimed at delaying cellular senescence by manipulation of the telomere/telomerase complex may be of benefit.
